# Targeting the cutaneous microbiota in atopic dermatitis: ‘A new hope’ or ‘attack of the CoNS’?

**DOI:** 10.1002/ctm2.865

**Published:** 2022-05-11

**Authors:** Chia‐Yu Chu

**Affiliations:** ^1^ Department of Dermatology National Taiwan University Hospital and National Taiwan University College of Medicine Taipei Taiwan

**Keywords:** AD, biofilm, microbiome, *Staphylococcus aureus*, *Staphylococcus epidermidis*

## Abstract

Although evidence showing that *Staphylococcus aureus (S. aureus)* is directly causative of atopic dermatitis (AD) is still lacking, there is evidence that *S. aureus* abundance is associated with disease flares and therapeutic responses. Patients receiving ATx201 OINTMENT 2% twice‐daily had a significant reduction in the abundance of *S. aureus* and increasing Shannon diversity of skin microbiome compared to vehicle after seven days. A small molecule with a narrow‐spectrum effect, especially on *S. aureus*, might be an attractive alternative for the treatment of AD.

1

Atopic dermatitis (AD) is a disease characterised by itchy and inflamed skin frequently associated with skin infections and has been clearly shown to have an altered skin bacterial flora when compared to non‐AD subjects.^1^ A recent meta‐analysis reported the prevalence of *Staphylococcus aureus (S. aureus)* carriage by patients with AD was 70% on lesional skin compared with 39% on non‐lesional skin of the same patients or skin of healthy controls.^2^, ^3^ The rate of *S. aureus* colonisation was related to disease severity.^3^

Although evidence showing that *S. aureus* is directly causative of AD is still lacking, there is evidence that *S. aureus* abundance is associated with disease flares and therapeutic responses,^4^ and *S. aureus* colonisation can drive AD‐like disease in mice.^5^

Coagulase‐negative staphylococci (CoNS) are a heterogeneous group of nearly 40 species that compose the majority of the Staphylococcus genus.^6^ Some CoNS species are able to fight against pathogens. Several previously unknown and potent anti‐*S. aureus* molecules have been discovered to be produced by skin CoNS species, such as *S. epidermidis*, *S. hominis* and *S. lugdunensis*.^2^, ^7^ The antimicrobial activity was identified as antimicrobial peptides (AMPs) produced by CoNS species including *S. epidermidis* and *S. hominis*. These AMPs were strain‐specific, highly potent and synergised with the human AMP LL‐37 that could selectively kill *S. aureus*.^1^ Some recent studies also suggest that topical application of commensal organisms such as *S. hominis* or *Roseomonas mucosa* could reduce AD severity, which supports an important role for commensals in decreasing *S. aureus* colonisation in patients with AD.^2^ Another study also showed that the human commensal *S. caprae* may compete with *S. aureus* by inhibiting quorum sensing. Through signal interference, *S. caprae* reduces methicillin‐resistant *S. aureus* burden in both skin colonisation and infection.^6^

In this issue of Clin Transl Med, Weiss et al.^8^ investigated the potency and the spectrum of ATx201 (niclosamide) in pre‐clinical models and further analysed its propensity for resistance evolution. The impact of ATx201 OINTMENT 2% on the skin microbiome and load of *S. aureus* was then assessed in a clinical trial in patients with mild‐to‐severe AD. They found that ATx201 has a narrow distribution of minimal inhibitory concentration (0.125–0.5 μg/ml) consistent with its mode of action – targeting the proton motive force effectively stopping cell growth. In a Phase II trial of 36 patients with mild‐to‐severe AD, they showed that patients receiving ATx201 OINTMENT 2% twice daily had a significant reduction in the abundance of *S. aureus* and increasing Shannon diversity of skin microbiome compared to vehicle after 7 days.^8^ However, the impact of ATx201 on the healthy skin microbiota and specificity of ATx201's effect to *S. aureus* remains to be fully elucidated. Accordingly, higher‐resolution metagenomics and longer treatment duration such as 28, 42 or 84 days are still needed along with approaches to quantitate whether increases in Shannon diversity result primarily from a reduction in *S. aureus* or a regrowth of the commensal microbiota. Furthermore, direct evidence of clinically significant improvement is still lacking.

Microbiome therapies are emerging therapies with the objective to reduce the abundance of *S. aureus* and increase microbial diversity by introducing one or more beneficial commensal strains. It was reported that transplantation of *S. hominis* and *S. epidermidis* strains reduced the bacterial load of *S. aureus* on the skin of AD patients, through exerting antimicrobial activity towards *S. aureus*.^1^ The presence of Esp‐secreting *S. epidermidis* was also shown to eliminate the nasal colonisation of *S. aureus* by diminishing the biofilm formation of *S. aureus*.^9^ While therapies based on addition of living bacteria represent a promising therapeutic approach, it remains unclear whether the use of transplants of specific commensal bacteria can eliminate resistant strains on the human AD skin. Long‐term stability of using commensal microorganism is another concern.^8^

*S. epidermidis* has commonly been regarded as a beneficial skin microbe to against *S. aureus*. However, a recent study^10^ found that the overabundance of *S. epidermidis* on some atopic patients can act similarly to *S. aureus*. Some strains of *S. epidermidis* may cause skin barrier damage and inflammation through secretion of the cysteine protease EcpA, and that this correlated with human disease severity.^10^ These findings suggest that *S. epidermidis* may shift from a beneficial commensal to a deleterious pathogen similar to *S. aureus* in the permissive growth conditions of AD skin. Some unique CoNS strains, such as *S. hominis* A9 and C5, are able to inhibit the *S. epidermidis* accessory gene regulator (agr) quorum sensing system and thus prevent *S. epidermidis*‐induced skin damage.^10^ *S. hominis* A9 has been shown to have the capacity to kill *S. aureus*, whereas *S. hominis* C5 synthetic autoinducing peptide was shown to inhibit the *S. aureus* agr system.^10^ Therefore, such commensal CoNS strains might be potential therapeutic tools to reduce *S. aureus* colonisation and both *S. aureus* and *S. epidermidis* toxin production in AD. The above findings indicate the complexity of the interactions between commensal CoNS and deleterious *S. aureus* and suggest that using *S. epidermidis* as the treatment for dysbiosis in AD may further damage the skin and cause inflammation. Thus, a small molecule with a narrow‐spectrum effect, especially on *S. aureus*, might be an attractive alternative for the treatment of AD.

## CONFLICT OF INTEREST

None.
